# Acute Supratentorial Ischemic Stroke: When Surgery Is Mandatory

**DOI:** 10.1155/2014/624126

**Published:** 2014-01-14

**Authors:** Gabriele Ronchetti, Pier Paolo Panciani, Roberto Stefini, Giannantonio Spena, Marco Maria Fontanella

**Affiliations:** Department of Neuroscience, Neurosurgery Unit, University of Brescia, Piazzale Spedali Civili 1, 25100 Brescia, Italy

## Abstract

Acute occlusion of middle cerebral artery (MCA) leads to severe brain swelling and to a malignant, often fatal syndrome. The authors summarize the current knowledge about such a condition and review the main surgical issues involved. Decompressive hemicraniectomy keeps being a valid option in accurately selected patients.

## 1. Introduction

Massive brain swelling may occur in up to 10% of cerebral ischemic strokes [1]. In these patients the clinical presentation usually starts with focal signs (motor weakness, speech disturbances, and hemianopsia) and progresses with a decline of consciousness (drowsiness, stupor) until brainstem dysfunction is evident (pupillary dilation, coma, and fatal systemic decompensation). Despite optimal medical management this condition may lead to death in 70–80% of cases [2, 3], and those who survive experience severe neurological deficits. Because of the grim prognosis, this condition has been termed “malignant” cerebral infarction.

The etiology is the occlusion of a large vessel, primarily the internal carotid (ICA) or the middle cerebral artery (MCA). A shift of the ischemic tissue rather than raised intracranial pressure (ICP) is the most likely responsible for the initial decrease in consciousness [4, 5]. Several other satellite reactions are involved in an inexorable pathogenetic cascade, including disturbances of microvascular tone, endothelial cell swelling, and activation of platelets, leucocytes, and coagulation [6].

In this review, we analyze the main clinical issues of malignant supratentorial cerebral infarction and summarize the key surgical issues commonly involved in its management.

## 2. Patients at Risk

At the onset, the clinical presentation of “malignant” cerebral infarction is no much different than any other acute ischemic stroke in the same distribution, although focal signs and symptoms tend to be severe from the outset. Massive brain swelling develops over time. Malignant evolution is more common in younger patients [3], likely due to the lack of atrophy and less tolerance to accommodate changes in brain volume.

Decompressive surgery seems to be more effective if performed earlier rather than later. Thus, there is great interest in identifying clinical, laboratory, and imaging findings able to predict which patients are going to develop a malignant infarction. The dosage of S100B, an astroglial protein released after neuronal injury, is a potentially useful laboratory tool. At 24 hours from clinical onset its serum value of 1.03 mcg/L has 94% sensitivity and 83% specificity to detect massive cerebral edema [7]. Unfortunately at this stage, this tool is not available in every hospital and its predictive value needs to be validated in larger studies.

Imaging studies are the mainstay for identification of people at higher risk for malignant infarction among the ischemic stroke population. Brain computed tomography (CT) is routinely performed for first and later controls. The earliest warning signs for developing malignant infarction include involvement of an area larger than 50% of the MCA territory and an infarct extending also to the anterior or posterior cerebral artery territories. A midline shift >10 mm, effacement of subarachnoid spaces, and attenuation of corticomedullary differentiation are also related to higher risk of severe deterioration [8], but they usually occur later, when a malignant syndrome is already in progress. The intravenous injection of contrast medium with elaboration of its distribution (perfusion-CT) entails higher diagnostic accuracy of ischemic areas and an even earlier detection of patients at higher risk. A drop in cerebral perfusion of more 66% is related to a likely malignant evolution [9].

Magnetic resonance imaging is another helpful exam, which in ischemic stroke can be used for prognostic purposes within few hours of clinical onset. Its sensitivity is higher than CT and it is more likely to show changes at earlier time points than CT scan. On diffusion weighted images (DWI) an ischemic area of at least 145 mL strongly predicts a massive cerebral infarction [10, 11]. Moreover, DWI ischemic volumes larger than 210 mL were found related to a 100% mortality in patients without surgical treatment [12].  Table 1 summarizes the main instrumental clues to detect ischemic patients at higher risk.

## 3. Intracranial Pressure (ICP) Monitoring

The first systematic use of a continuous ICP monitoring was historically made among patients with brain tumors [13]. This monitoring was then tested and applied to other conditions, and further improvements in technology and technique [14] contributed to its worldwide diffusion. Although widely accepted as useful tool in the management of patients with severe head injuries, the role of ICP monitoring in patients with large cerebral infarctions is controversial.

It is straightforward that at final stages the pressure inside the skull of patients with large cerebral infarction is probably high. Anyway, a pressure increase limited to the infarcted and immediately adjacent areas could happen, leading to neurological worsening and even death despite no spread of intracranial hypertension [15]. Undisputed poor prognosis predictors as CT uncal herniation and anisocoria sometimes occur without an overall ICP raise is detected [5]. The measurement may also be influenced by the device used (solid-state or fluid-filled) as well as by its location (subdural, intraparenchymal, intraventricular; ipsilateral or contralateral to ischemia) [16].

In patients with cerebral infarction, it is currently possible to have a clear estimate of the initial ischemic damage and to early detect the well-known possible clinical evolutions. Observing the clinical status, mainly by neurological examination in awake patients and by radiological studies in sedated, leads to more useful suggestions about the proper management rather than looking for intracranial hypertension, which may also never come. The latter for sure may occur, but it should be avoided rather than measured. In this scenario, there are no absolute recommendations for a routine use of intracranial pressure monitoring [5, 15], as it can be considered a not risk-free procedure which cannot actually influence the final clinical management. In fact, patients with malignant infarction syndrome have to be aggressively treated, regardless of any quantitative element we could measure.

## 4. Considering a Surgical Option

Despite standard treatment, patients with massive cerebral infarction get worse, usually within 24–48 hours from the clinical onset [17]. This generally means drowsiness, altered consciousness, motor dysfunctions, hypertension, bradycardia, and in the unlucky cases progressive decline with dilated pupils and respiratory failure [3, 17]. Osmotherapy, buffers, sedation, mannitol, hyperventilation [18, 19], and more recently hypothermia [20–22] are the intensive care treatments which can be applied in patients with clinical worsening due to ischemic tissue swelling. Unfortunately, they represent only short-lived interventions and temporizing measures which just slow the inexorable development of further deterioration from tissue displacement and brain stem shift [2, 3, 23, 24].

Surgery conversely can be very effective with adequate indications; the reasonable operative treatment in massive cerebral infarction is decompressive hemicraniectomy. The goal of such removing of a part of the cranial vault is to reduce the pressure of the swollen ischemic tissue and to save the brain that is still viable. Several animal studies demonstrated the biological effects of this surgical procedure, as the improvement of the overall cortical perfusion [25, 26] and the reduction of apoptosis in the ischemic border zone [27]. To not consider the surgical option leads to missed opportunities of successful treatment [28], but on the other hand not all the patients with the above requisite may really benefit from the intervention.

## 5. Best Conditions for Surgery

Timing is an important issue to consider when evaluating ischemic patients for surgery. Surgery cannot resurrect dead neurons. The warning signs we mentioned do not have to all occur before the neurosurgeon is involved because this evident brainstem suffering would lead to poor prognosis despite any effort. Decompressive hemicraniectomy should be performed within 48 h of stroke [29, 30], before brainstem dysfunction is patent. The requisite for the surgical indication is an even initial worsening in patients with verified massive cerebral infarction, and clinical trials failed to demonstrate benefits by prophylactic ultra-early surgery as no differences were found between patients treated at 24 and 48 hours from stroke onset [30–32]. Anyway the possible occurrence of a radiological worsening despite a stable clinical status keeps being an unresolved issue for indication to decompress.

The second factor that the neurosurgeon has to consider is the patient prestroke condition, which is a good predictor of the chance of survive and mostly of the quality of life in case of surgical intervention [33, 34]. In the available clinical trials the patient age resulted in one of the most reliable outcome indicators. Generally, patients older than 60 years are not ideal candidates for surgical decompression [35, 36], as they possess a lower neuronal plasticity and also frequently have more vascular risk factors and comorbidities. Even in young patients with severe hypertension, cardiac failure, pulmonary embolism, and other analogous unfavorable diseases the hemicraniectomy might result highly risky and less effective. Conversely also in older patients with a good antecedent condition hemicraniectomy seems somehow to improve the prognosis [37]. As not univocal data result from literature it is suggested to not routinely perform surgical decompression in stroke patients older than 60 years at least in really well-selected cases. The effects of hemicraniectomy in patients aged over 65 years will be anyway assessed in the ongoing Destiny II trial.

Surgery can be safely performed even after intravenous tissue plasminogen activator administration for thrombolysis [38, 39]. The side of the stroke does not seem to affect the vital status after surgical decompression, so it should not influence the choice to operate [32, 40]. However, the family should be informed about the likely chance for the patient to survive but with severe speech disturbances. Family in turn may provide useful information concerning the patient's wishes [41].

**Figure 1:**
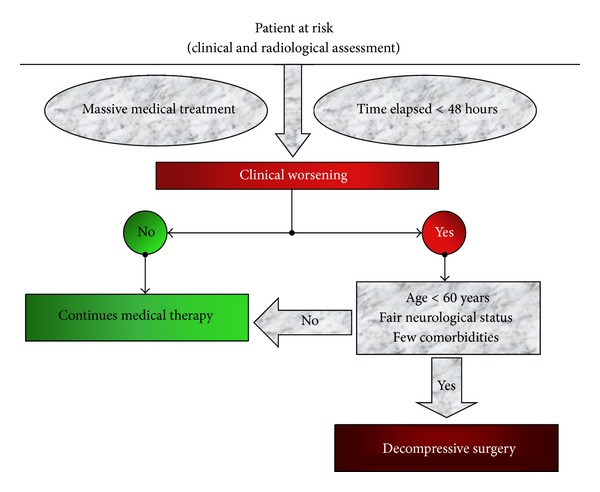
Flowchart for best conditions for surgical decompression in patients with massive cerebral (MCA) infarction.

Figure 1 shows the flowchart for the best conditions for surgical decompression.

## 6. Decompressive Technique

**Figure 2:**
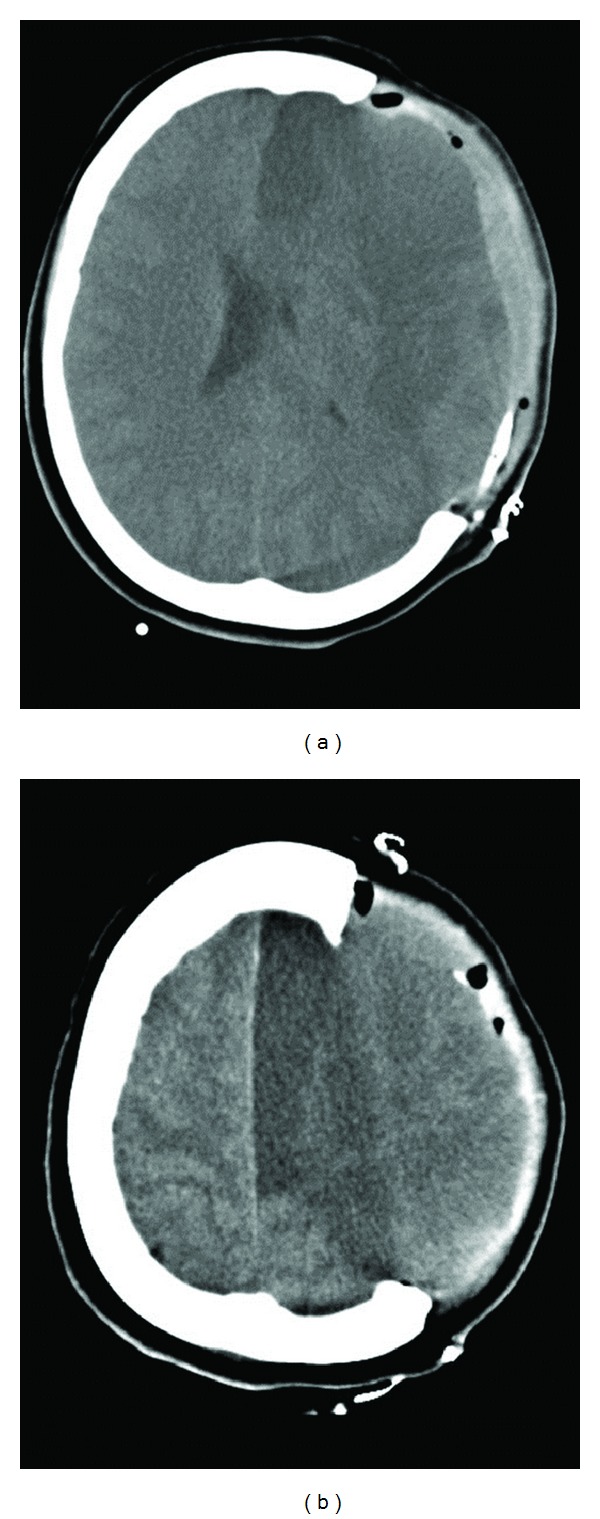
CT scan in a 54-year old female patient who underwent decompressive hemicraniectomy for a large MCA infarction. Note the large bone removal (a) and the left hemisphere occupying this new free space (b).

The skin incision can be made as a big question mark or a midline leaf-spring. Some surgeons advocate resection of the temporal muscle and fascia to allow a maximum decompression [42], but this is not commonly performed. The craniotomy should include the frontal, parietal, and temporal bones and its anteroposterior length should not be inferior to 12 cm (Figure 2(a)); larger openings up to 14 cm or more are thought to allow an even better pressure relieve [43]. Particular attention has to be paid to decompression of the basal temporal area, as it represents a critical compartment with close relationship with the brainstem. In order to gain additional room, the dura mater is commonly opened as well. It can then be enlarged with a biological or synthetic substitute or left patent, just covered by hemostatic material for a faster closure [44]. The cerebral tissue itself should be completely preserved at surgery for recovery of the not deadly damaged areas, which may be not distinguishable from the infarction itself (Figure 2(b)).

The bone flap can be preserved in a subcutaneous pocket overlying the abdomen, but this leads to partial reabsorption in the following weeks, longer operation times, and additional risks related to the additional wound. A valid and currently prevailing alternative is to store the bone frozen (−80°C) in a sterile box. The possible complications of decompressive craniectomy are surgical site infections, hemorrhagic troubles and extra-axial fluid collections, hydrocephalus, and the so-called sinking flap syndrome.

## 7. What to Expect from Surgery

The surgical decompression is not a panacea and it is clear that patients will never go back to being intact as before the ischemia. The survival rates are for sure higher in those who undergo surgery [12, 45–47]. Within the survivors hemicraniectomy reduces the occurrence of a vegetative state and increases the chances of a functional independence [32, 48]. Anyhow in some patients the quality of life remains low [34]. A residual motor dysfunction is the rule, and speech disturbances almost always affect patients with a dominant side infarction. Depression and cognitive impairment are common as well [49].

The criteria for surgical indication mean a selection of patients who likely will have less postoperative disabilities. Living with a severe neurological impairment may appear more acceptable in some cultures, and inhumane in others. A recent review anyway concluded that the vast majority of operated patients do not regret having undergone surgery [50].

## 8. Cranioplasty

Once the clinical status and radiological imaging are compatible, the bone flap can be repositioned to restore structural integrity, protect the brain, and reinstate intracranial pressure. It is not exceptional that after cranioplasty the patients show some clinical improvement [51, 52]. The need to primarily use different materials than the autologous bone to cover the skull defect is uncommon in patients decompressed for ischemic stroke. The perfect timing for cranioplasty after hemicraniectomy is debated, and in the literature do articles specific for patients who underwent surgery for malignant cerebral infarction not exist. It can anyway be deduced from more general studies that the bone flap can be usually repositioned within 5 to 12 weeks [51, 53–55]. Infections and persistent parenchymal herniation through craniectomy necessarily require longer waiting times for this second surgical operation.

The cranioplasty clearly may have complications: infection and extra-axial hematoma formation are the most common [43] followed by hydrocephalus. Postoperative intracerebral infarction is fortunately a rare happening [51, 56]. Sudden brain swelling and late bone resorption are possible complications as well.

## 9. Conclusions

Some patients with ischemic stroke develop a diffuse and progressive cerebral swelling leading to a frequently fatal condition named malignant cerebral infarction syndrome. It is important to detect early the cases at higher risk for that, in order to intervene before definitive massive brain injury occurs. For this purpose both radiological and clinical observations are helpful; intracranial pressure monitoring is not indispensable.

Medical treatments regularly fail in patients with neurological decline for massive cerebral infarction, and the surgical option can be therein considered. Decompressive hemicraniectomy is a life-saving procedure which also leads to better functional outcomes. A large unilateral craniotomy with duraplasty is performed, and the brain parenchyma is spared. The patients who mostly benefit from such intervention are the younger and previously healthier, treated within a narrow period from the clinical deterioration. In any case, the persistence of postoperative deficits is the rule. A complete and honest explanation of the utility, likely result, and limits of the surgical operation should always be given to the relatives of the patient candidate for decompression. Cranioplasty can be usually performed within few weeks, when both clinical status and radiological status are favorable.

The lack of class I evidences about most of the discussed issues is constraining and the need for further studies on medical and surgical effectiveness is undoubted. Anyway at present time the above management flowchart for these patients may be suggested, where the surgical intervention should be considered an extreme but not senseless option once the more suitable cases are selected.

## Figures and Tables

**Table 1:** Summary of the main instrumental clues to detect ischemic patients at higher risk for a malignant supratentorial (MCA) infarction.

Exam	Result
Plasmatic S100B protein dosage	>1.03 mcg/L
CT scan/perfusion-CT	Area compromised > 50% MCA territory
	Extension to anterior or posterior territories
	Perfusion drop > 66%
MRI/DWI	Ischemic area > 145 mL, even at early stages
